# Potential Role of Mineralocorticoid Receptor Antagonists in Nondiabetic Chronic Kidney Disease and Glomerular Disease

**DOI:** 10.2215/CJN.0000000000000540

**Published:** 2024-07-22

**Authors:** Teena Zachariah, Jai Radhakrishnan

**Affiliations:** Columbia University Irving Medical, New York, New York

**Keywords:** clinical nephrology, glomerular disease, nephrology, proteinuria, glomerular diseases

## Abstract

Glomerular disease is a leading cause of CKD and ESKD. Although diabetic kidney disease is the most common cause of glomerular disease, nondiabetic causes include malignancy, systemic autoimmune conditions, drug effects, or genetic conditions. Nondiabetic glomerular diseases are rare diseases, with a paucity of high-quality clinical trials in this area. Furthermore, late referral can result in poor patient outcomes. This article reviews the current management of nondiabetic glomerular disease and explores the latest developments in drug treatment in this area. Current treatment of nondiabetic glomerular disease aims to manage complications (edema, hypertension, proteinuria, hyperlipidemia, hypercoagulability, and thrombosis) as well as target the underlying cause of glomerular disease. Treatment options include renin-angiotensin-aldosterone system inhibitors, statins/nonstatin alternatives, loop diuretics, anticoagulation agents, immunosuppressives, and lifestyle and dietary modifications. Effective treatment of nondiabetic glomerular disease is limited by heterogeneity and a lack of understanding of the disease pathogenesis. Sodium-glucose cotransporter-2 inhibitors and nonsteroidal mineralocorticoid receptor antagonists (ns-MRAs, such as finerenone), with their broad anti-inflammatory and antifibrotic effects, have emerged as valuable therapeutic options for a range of cardiorenal conditions, including CKD. ns-MRAs are an evolving drug class of particular interest for the future treatment of nondiabetic glomerular disease, and there is evidence that these agents may improve kidney prognosis in various subgroups of patients with CKD. The benefits offered by ns-MRAs may present an opportunity to reduce the progression of CKD from a spectrum of glomerular disease. Several novel ns-MRA are in clinical development for both diabetic and nondiabetic CKD.

## Introduction

Glomerular diseases, both diabetic kidney disease and nondiabetic glomerular disease, are common causes of CKD and ESKD, with some reports suggesting the incidence of CKD and ESKD are increasing worldwide.^1,2^ Although diabetic kidney disease is the most common cause of glomerular disease, nondiabetic glomerular diseases also contribute significantly to CKD/ESKD.^2,3^ Nondiabetic glomerular disease includes primary forms (such as IgA nephropathy, FSGS, membranous nephropathy) and glomerular disease secondary to systemic disease, including lupus nephritis, and ANCA-associated vasculitis.^3–6^ Aside from being rare diseases, individual glomerular diseases are highly heterogenous, making recruitment for clinical trials difficult.^7–9^ Moreover, in the absence of screening programs for kidney disease, nondiabetic kidney disease is associated with late referral, which in turn may result in poorer patient-related outcomes.^10^ Finally, there is an increasing rate of nondiabetic kidney disease being diagnosed through biopsy in patients with diabetes, which confounds accurate classification of glomerular disease.^11,12^

Here we review the current management of nondiabetic glomerular disease and consider the associated unmet need for affected patients, as well as explore the latest developments in drug treatment. The evolving drug class of mineralocorticoid receptor antagonists (MRAs) is of particular interest, with evidence of improved kidney prognosis in various subgroups of patients with CKD.

## Epidemiology of Nondiabetic Glomerular Disease

Discounting diabetic nephropathy, incidence rates of glomerular disease in adults are between 0.2 and 2.5 per 1000 population depending on the type of glomerular disease (rates based on database search published in 2011); these are classified as rare diseases.^4^ Among the most common nondiabetic glomerular diseases are IgA nephropathy, membranous nephropathy, and FSGS.^13^ Globally, the incidence of IgA nephropathy is higher in Asian versus non-Asian individuals and is higher in male patients than in female patients.^14^ Primary membranous nephropathy occurs more in White men older than age 40 years.^15^ Estimates of prevalence and/or incidence are confounded by difficulties in classifying glomerular disease, *e*.*g*., nondiabetic glomerular disease superimposed on diabetic glomerular disease.^13^ There are also important gender differences, *e*.*g*., IgA nephropathy occurs in almost 65% of male patients versus approximately 35% female patients with glomerular disease in some geographic areas, whereas lupus nephritis occurs in <20% of male patients versus approximately 80% of female patients with glomerular disease.^16^ Those living with chronic hypertension are also at risk of progressive kidney failure in the form of hypertensive nephrosclerosis.^17,18^ This syndrome is more common in older age and in people with poorly controlled moderate to severely high BP.^17,18^

## Management of Nondiabetic Glomerular Disease and the Current Unmet Need

Assessment of kidney function on the basis of eGFR, urine microscopy, and quantification of proteinuria is key to indicate the presence of glomerular disease.^3^ Treatment strategies include the management of complications such as hypertension (lifestyle modifications, renin-angiotensin-aldosterone system inhibitors [RAASis], MRAs, other antihypertensive agents), edema (loop diuretics, restricted dietary sodium intake), hyperlipidemia (lifestyle modifications, statin, or a nonstatin alternative), hypercoagulability, and thrombosis (anticoagulation agents).^3^ Proteinuria reduction strategies include the use of RAASis^3^ with evidence to suggest that sodium-glucose cotransporter-2 inhibitors (SGLT2is) may also be beneficial.^19,20^ Treatment may also target the underlying cause of glomerular disease, *e*.*g*., BP control, and treatments for infections such as hepatitis and HIV, or removal of potentially nephrotoxic agents, such as bisphosphonates.^3,17,18^ Specific treatment regimens for common glomerular diseases are listed in Table 1.

**Table 1:** Overview of common glomerular diseases and pharmaceutical agents used for their management

Disease	Treatment
**Nephrotic syndrome**	
Minimal change disease^3^	• Glucocorticoids • Alternative: cyclophosphamide, calcineurin inhibitors, mycophenolate mofetil/sodium mycophenolate+reduced-dose glucocorticoids, rituximab
Membranous nephropathy^3^	• Rituximab±calcineurin inhibitor • Calcineurin inhibitor±glucocorticoids • Cyclophosphamide+glucocorticoids
FSGS^3^	• Glucocorticoids • Calcineurin inhibitors
**Nephritic syndrome (nephrotic-nephritic syndrome)**	
Anti-GBM antibody disease^3^	• Cyclophosphamide • Glucocorticoids • Plasmapheresis
ANCA-associated vasculitis^3,87^	• Glucocorticoids+cyclophosphamide or rituximab (induction)+avacopan • Rituximab or azathioprine+glucocorticoids (maintenance)
Immune complex GN	
*Infection-related GN^3^*	• Antibiotics for underlying bacterial infection • Use of glucocorticoids and immunosuppression unproven and generally should not be used
*Lupus nephritis^3^*	• Hydroxychloroquine • Induction therapy: glucocorticoids+cyclophosphamide or mycophenolic acid analogs • Calcineurin inhibitors (including voclosporin) or B-lymphocyte targeting biologics (belimumab) may be added • Maintenance therapy: tapering glucocorticoids with mycophenolic acid analogs or azathioprine or mizoribine (Japanese patients)
*IgA nephropathy^3,22,23^*	• RAAS inhibitors • Glucocorticoids (for patients at high risk of progressive CKD despite maximal supportive care) • Mycophenolate (for Chinese patients in whom glucocorticoids are being considered) • Hydroxychloroquine (in Chinese patients who remain at high risk of progression despite supportive care) • Targeted release budesonide • Sparsentan
*C3 glomerulopathy^3^*	• Mycophenolate mofetil+glucocorticoids • Alternative: eculizumab
Monoclonal gammopathy of renal significance^88–90^	• Rituximab, daratumumab, alkylating agents (such as cyclophosphamide), immunomodulatory drugs, autologous stem cell transplant
Hypertensive nephrosclerosis^91^	• RAASi
Diabetic kidney disease^28,29^	• RAASi, SGLT2is, finerenone

GBM, glomerular basement membrane; RAAS, renin-angiotensin-aldosterone system; RAASi, renin-angiotensin-aldosterone system inhibitor; SGLT2i, sodium-glucose co-transporter-2 inhibitor.

Effective treatment of nondiabetic glomerular disease is limited by heterogeneity and a lack of complete understanding of the underlying pathogenesis. For example, glucocorticoids are the mainstay of treatment for patients with IgA nephropathy at high risk of progressive CKD despite maximal supportive care, yet their long-term adverse effects and requirement for high dosage make their use questionable. Furthermore, there are conflicting reports about the long-term effect on kidney outcomes. Even with the approval of two novel agents, sparsentan and targeted-release budesonide, both of which reduce proteinuria and stabilize the eGFR,^21–23^ it is unclear whether currently available therapies will, in the long term, slow CKD progression in IgA nephropathy. Other therapeutic targets being investigated include B cell and complement.^24^ By contrast, other glomerular disease conditions such as minimal change disease, which are expected to remit rapidly with immunosuppression, or some infection-related glomerulonephritides (treatment through removal of the inciting factor) will likely not need chronic therapy in the absence of persistent proteinuria or CKD.^3^ There is an increasing awareness of the role of aldosterone in the pathogenesis of CKD, and higher serum aldosterone levels among individuals with CKD are independently associated with an increased risk for kidney disease progression, irrespective of concomitant diabetes.^25^ Furthermore, in patients taking RAASis, aldosterone levels may not be suppressed (so-called aldosterone breakthrough).^26,27^ A RAASi may be used in combination with a steroidal MRA because of the complementary proteinuria-lowering effects or in combination with a nonsteroidal (ns-)MRA (*e*.*g*., finerenone) in CKD associated with type 2 diabetes.^3,28–30^

## MRAs

**Figure 1:**
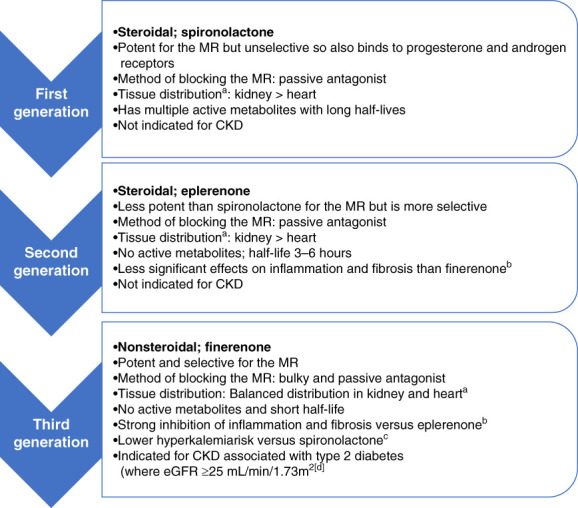
**Three phases of evolution of MRAs.**^31–40,69^ ^a^In rodents. ^b^In a mouse model when compared with equinatriuretic doses of comparator. ^c^On the basis of phase 2 study (ARTS) with spironolactone as active comparator. ^d^eGFR on the basis of Kerendia (finerenone) FDA label, 2022. ARTS, mineralocorticoid receptor antagonist tolerability study; FDA, US Food and Drug Administration; MR, mineralocorticoid receptor; MRA, mineralocorticoid receptor antagonist.

There are two types of MRA in current use: steroidal MRAs and ns-MRAs. Figure 1 gives an evolutionary overview of the first-generation, second-generation, and third-generation MRAs.^31–40^ Steroidal MRAs are indicated for the treatment of resistant hypertension^37,38^; however, the potential application of these drugs in hypertensive nephrosclerosis has not been fully evaluated in clinical trials. The Kidney Disease Improving Global Outcomes (KDIGO) guidelines currently suggest that a steroidal MRA may be used as an alternative to a RAASi if an angiotensin-converting enzyme inhibitor (ACEi) or angiotensin II receptor blocker (ARB) cannot be used for management of hypertension and proteinuria reduction in glomerular disease.^3^ KDIGO also recommends conducting randomized controlled trials (RCTs) to investigate the therapeutic strategy of adding an MRA to a RAASi in the treatment of nondiabetic proteinuric kidney diseases.^3^ Furthermore, a phase 3 clinical trial looking at the efficacy and safety of the ns-MRA finerenone in adults with nondiabetic CKD (Finerenone In NonDiabetic-CKD; NCT05047263) is ongoing. However, there are no ongoing or recently completed phase 3 clinical trials with steroidal MRAs specifically in patients with nondiabetic proteinuric kidney disease.

## MRAs: Mode of Action and Class Evolution

The mineralocorticoid receptor (MR) is expressed in many tissues (kidney, heart, colon, brain) and cell types (*e*.*g*., immune cells and fibroblasts).^31,41^ Physiologically, the MR is involved in fluid, electrolyte, and hemodynamic homeostasis, as well as tissue repair. The overlapping expression and functional interactions of the MR with glucocorticoid receptors are critical to maintaining homeostatic balance.^42^ From a pathophysiologic standpoint, MR overactivation causes inflammation and fibrosis in cardiorenal tissues and is therefore an important aspect of cardiorenal disease development and advancement.

MRAs inhibit the action of aldosterone at the MR, preventing receptor activation, which prevents remodeling and decreases inflammation.^43,44^ Critically, MRAs also reduce proteinuria,^45^ which is an established risk factor for the progression of kidney disease in patients with diabetic and nondiabetic nephropathy^46^; indeed, proteinuria (specifically albuminuria) is used in CKD classification.^3,28,29^ Although it is important to note that although all currently approved MRAs have shown antiproteinuric effects, only the ns-MRA finerenone is indicated for use in CKD associated with type 2 diabetes.^36^

The first MRA investigated for therapeutic potential was spironolactone, which is steroidal and nonspecific in its action affecting androgen and progesterone receptors.^37^ Spironolactone was initially approved by the US Food and Drug Administration (FDA) in 1960 and is indicated for the treatment of New York Heart Association Class III–IV heart failure and reduced ejection fraction, hypertension, edema in patients with cirrhosis, and primary hyperaldosteronism.^37^ However, in addition to the nonspecific adverse effects noted in the pivotal Randomized Aldactone Evaluation Study clinical trial in symptomatic/severe heart failure (gynecomastia or breast pain significantly more frequent versus placebo),^47^ postmarketing data revealed a higher risk of hyperkalemia associated with spironolactone treatment, raising concern over its risk–benefit profile.^48^ A more selective steroidal MRA, eplerenone, was approved in 2002 and is indicated for use in improving survival of stable patients with heart failure after acute myocardial infarction and for hypertension.^38^

The latest development is the approval of the ns-MRA finerenone, which is the first third-generation MRA authorized in the United States for use in reducing the burden of cardiorenal disease. It is selective in its action and is more potent than eplerenone.^31–33^ Finerenone is indicated to reduce the risk of sustained eGFR decline, ESKD, cardiovascular death, nonfatal myocardial infarction, and hospitalization for heart failure in adult patients with CKD associated with type 2 diabetes.^36^

## Experience of MRAs in Diabetic Glomerular Disease

The renin-angiotensin system has been a therapeutic target for the management of diabetic glomerular disease for decades.^49,50^ Use of steroidal MRAs (*e*.*g*., spironolactone) in the setting of cardiovascular indications has revealed kidney protective effects, highlighting the intrinsic links between heart and kidney outcomes.^51^ A subanalysis of a phase 3 clinical trial of spironolactone in heart failure showed the antiproteinuria effects of this drug (39% placebo-adjusted reduction in albuminuria).^52^ The results from a phase 4 study of high-dose eplerenone in people with type 2 diabetes and high cardiovascular risk also showed an encouraging antiproteinuric effect (34% reduction in albuminuria versus placebo).^53^ However, the results of a pooled analysis of steroidal MRA clinical trials showed that although these drugs reduce heart failure hospitalizations and mortality across a wide range of patients with kidney dysfunction (based on baseline eGFR), these benefits are much reduced and have associated safety concerns in patients who had more severe kidney dysfunction at baseline, particularly those with eGFR ≤30 ml/min/1.73 m^2^.^54^ There is an absence of phase 3 outcome data with steroidal MRAs in the kidney space.

**Figure 2:**
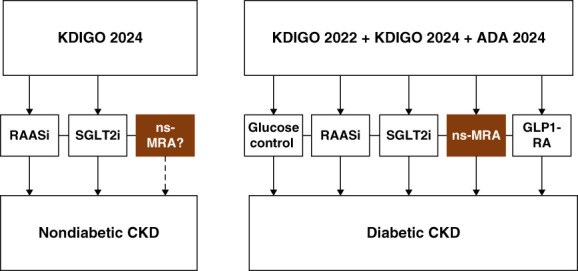
**Treatment pathway for ns-MRA use in diabetic CKD and potential ns-MRA use in nondiabetic CKD incorporating clinical practice guideline (graded) recommendations.**^28,29,108^ Initiation of SGLT2i therapy requires a pretreatment eGFR of ≥20 ml/min per 1.73 m^2^; initiation of an ns-MRA (currently only finerenone) therapy requires a pretreatment eGFR ≥25 ml/min per 1.73 m^2^ and normal serum potassium. ADA, american diabetes association; GLP1-RA, glucagon-like peptide-1 receptor agonist; KDIGO, Kidney Disease Improving Global Outcomes; ns-MRA, nonsteroidal mineralocorticoid receptor antagonist; RAASi, renin-angiotensin-aldosterone system inhibitor; SGLT2i, sodium-glucose co-transporter-2 inhibitor.

With the approval of finerenone for treating CKD associated with type 2 diabetes, focus has now shifted to the potential value of ns-MRAs. Table 2 gives an overview of MRA studies in CKD, including finerenone in CKD associated with type 2 diabetes. The FInerenone in reducing kiDnEy faiLure and dIsease prOgression in Diabetic Kidney Disease (FIDELIO-DKD) trial demonstrated that finerenone, on a background of ACEi/ARB treatment, significantly reduced the risk of a primary composite kidney endpoint (time to onset of kidney failure, sustained ≥40% decline in eGFR from baseline, or death from kidney causes) compared with placebo in patients with CKD and type 2 diabetes.^55^ Overall, the frequency of adverse events was similar in the finerenone and placebo groups, although the rate of hyperkalemia-related discontinuation of the study drug was higher than for placebo.^55^ In the FInerenone in reducinG cArdiovascular moRtality and mOrbidity in Diabetic Kidney Disease (FIGARO-DKD) trial, finerenone, on a background of ACEi/ARB treatment, significantly reduced the risk of a primary composite cardiovascular endpoint (composite of death from cardiovascular causes, nonfatal myocardial infarction, nonfatal stroke, or hospitalization for heart failure) compared with placebo in patients with CKD and type 2 diabetes.^56^ Although there was no significant effect of finerenone on the composite kidney endpoint (equivalent to the primary endpoint of FIDELIO-DKD; the secondary endpoint in this trial), it was associated with a significant 23% reduction in another kidney-specific composite endpoint that included a sustained ≥57% decline in eGFR from baseline.^56^ Safety findings were similar to FIDELIO-DKD.^55,56^ Hyperkalemia is included under the Warnings and Precautions section of the FDA label for finerenone (patients with decreased kidney function and higher baseline potassium levels being at increased risk), but this risk can be mitigated with monitoring of serum potassium levels and dose adjustments as needed.^36^ Potassium binders, such as patiromer and sodium zirconium cyclosilicate, may also be an option to treat hyperkalemia.^28,57^ The FIDELITY (FInerenone in chronic kiDney diseasE and type 2 diabetes: Combined FIDELIO-DKD and FIGARO-DKD Trial programme analYsis) analysis of data from both studies demonstrated the efficacy of finerenone in improving hard cardiovascular and kidney failure outcomes in a broad spectrum of patients with type 2 diabetes and CKD.^58^ A meta-analysis of FIGARO-DKD, FIDELIO-DKD, and five phase 2 RCTs further corroborated the kidney benefits of finerenone in patients with CKD and diabetes, demonstrating significant benefits over placebo in reducing urine albumin-to-creatinine ratio (UACR) and eGFR decline.^59^ Figure 2 shows a treatment pathway for ns-MRA use/potential ns-MRA use in diabetic and nondiabetic CKD. Esaxerenone is another ns-MRA that has been studied in patients with CKD associated with type 2 diabetes. The results from the phase 3 ESAX-DN study (esaxerenone in patients with type 2 diabetes and microalbuminuria), showed that esaxerenone added to RAASi therapy, significantly reduced UACR, increased the chances of albuminuria remission in some patients, and reduced the risk of albuminuria progression versus placebo.^60^ Esaxerenone is approved for use in Japan for hypertension^61^ but is not approved for use in CKD or hypertension in the United States.

**Table 2:** Summary of randomized controlled trials^a^ of mineralocorticoid receptor antagonist in nondiabetic CKD^b^ and diabetic (type 2 diabetes) CKD

Trial	Phase	Design	*N*	Key Efficacy Results/conclusions and Post-treatment Serum Potassium/incidence of Hyperkalemia
**Nondiabetic CKD** ^a^ ^,^ ^b^				
Spironolactone				
*Wang et al. 2013^92^*	—	Open-label, randomized trial of ACEi/ARB±spironolactone in patients with chronic glomerular disease	221 (39 with diabetic nephropathy)	Spironolactone+ACEi/ARB treatment markedly decreased urine protein levels No significant difference in serum potassium levels across both groups
*Bianchi et al. 2006^93^*	—	Randomized, open-label study of spironolactone in patients with nondiabetic CKD previously treated with ACEi/ARB	165 (all nondiabetic CKD)	After 1 yr, decrease in eGFR from baseline was lower in patients treated with spironolactone than in controls, and spironolactone caused a significant rise in serum potassium levels Low incidence of hyperkalemia
*Guney et al. 2009^94^*	—	Randomized preliminary study of ACEi/ARB±spironolactone in patients with nondiabetic CKD	30 (all nondiabetic CKD)	Spironolactone reduced both proteinuria and urinary TGF-*β*1 excretion Serum potassium levels increased with spironolactone in third month but not the sixth month; increases were not clinically significant
*Hammer et al. 2010^95^*	—	Single-center randomized, double-blind, placebo-controlled study of spironolactone+ACEi/ARB in patients with nondiabetic CKD	112 (all nondiabetic CKD)	Spironolactone+ACEi/ARB treatment significantly decreased albumin creatinine ratio Six patients had hyperkalemia, and one patient had serious hyperkalemia and discontinued spironolactone; all during open label run-in
*Abolghasmi and Taziki 2011^96^*	—	Randomized double-blind, placebo-controlled study of spironolactone+antihypertensives in patients with CKD and resistant hypertension	41 (CKD etiology not specified)	Spironolactone+antihypertensive treatment significantly reduced BP Full safety data nor overview included in the publication
*Edwards et al. 2010^97^*	2	Single-center, double-blind, randomized, placebo-controlled study of spironolactone+ACEi/ARB in patients with early-stage CKD	112 (all nondiabetic CKD)	Spironolactone exerts beneficial effects on LV function No safety data overview included in the publication
*Furumatsu et al. 2008^98^*	—	Open-label RCT of spironolactone+ACEi and ARB in patients with nondiabetic CKD	30 (all nondiabetic CKD)	Triple blockade with spironolactone+ACEi and ARB was more effective at reducing proteinuria than dual blockade (trichlormethiazide+furosemide) No safety data overview included in the publication
*Tylicki et al. 2008^99^*	—	Randomized, open-label, 2×2 crossover study of spironolactone+ACEi/ARB in patients with nondiabetic CKD	18 (all nondiabetic CKD)	Triple blockade with spironolactone+ACEi and ARB was significantly reduced proteinuria compared with dual blockade (ACEi+ARB) Serum potassium increased significantly (versus baseline) after triple blockage, but not after double blockade
*Chrysostomou et al. 2006^100^*	—	Randomized, double-blind, placebo-controlled study of ACEi±ARB and/or spironolactone in patients with proteinuria (and reduced serum creatinine)	41 (27 with diabetic nephropathy)	Spironolactone with an ACEi alone or in combination with an ARB resulted in a significant reduction in proteinuria Gynecomastia not reported; hyperkalemia not considered a clinical problem in the study
*Smolen and Nowicki 2006^101^*	—	Randomized study to compare low-dose spironolactone with hydrochlorothiazide in patients with chronic GN and persistent non-nephrotic proteinuria	16 (etiology not specified)	Low-dose spironolactone (+ACEi) reduces proteinuria independent of changes in BP Potassium levels were significantly increased during spironolactone treatment
*Tokunaga et al. 2008^102^*	—	Randomized, single-center study of ARBs±spironolactone in patients with stage 3–4 CKD	64 (etiology not specified)	Spironolactone in combination with an ARB is effective in slowing the progression of kidney insufficiency in patients with stages 3–4 CKD Increases in serum potassium with spironolactone, but no discontinuations because of hyperkalemia
Eplerenone				
*Boesby et al. 2013^103^*	3	Randomized, open label, parallel-group study of eplerenone or standard medication in patients with stage 3–4 CKD	54 randomized (no patients in eplerenone group had diabetic nephropathy; 27% had diabetes)	Add-on treatment with eplerenone did not significantly reduce carotid femoral pulse wave velocity Mild hyperkalemia was observed
*Ando et al. 2014^104^*	—	Double-blind, randomized, placebo-controlled trial of eplerenone in hypertensive patients with nondiabetic kidney disease who had received RAASis	314 (all nondiabetic CKD)	Addition of low-dose eplerenone to RAASis might have renoprotective effects through reduction of albuminuria No serious safety concerns were observed Serum potassium slightly but significantly increased with eplerenone versus placebo but no cases of hyperkalemia (potassium >5.5 mmol/L) in either group
*Minakuchi et al. 2020^66^*	—	Randomized placebo-controlled trial of eplerenone or placebo in patients with stage 2 and 3 CKD	141 (diabetic nephropathy in 14.2%)	At 24 and 36 mo, eGFR was significantly higher in the eplerenone group than in the placebo group The benefits were most pronounced in patients with high plasma aldosterone No serious safety concerns were observed Plasma potassium significantly increased with eplerenone versus placebo, but no serious hyperkalemia episodes reported
*Provenzano et al. 2022^71^*	—	Randomized, open-label, crossover trial of dapagliflozin and eplerenone alone and in combination in patients with CKD who had received ACEi/ARB	46 (69.6% had type 2 diabetes)	Robust additive UACR-lowering effect observed when combining dapagliflozin with eplerenone (a clinically relevant albuminuria reduction of 53% after 4 wk) Incidence of hyperkalemia less when eplerenone used in combination with dapagliflozin
*Boesby et al. 2011^105^*	4	Randomized, open-label, crossover trial comparing an 8-wk control period with an 8-wk period of eplerenone in patients with nondiabetic CKD	40 (all nondiabetic CKD)	Add-on eplerenone to stable antihypertensive treatment caused a 22% reduction in urinary albumin excretion versus a control period Potassium levels were increased during eplerenone treatment
*Cohen et al. 2011^106^*	—	Randomized study of eplerenone in patients with stage 1–3 CKD who had received ACEi/ARB	34 (etiology not specified)	There was a significant correlation between baseline arterial stiffness and decrease in proteinuria in patients receiving eplerenone+RAASi (congress abstract)
*Haykal et al. 2007^107^*	—	Randomized study of eplerenone in patients with stage 1–3 CKD and non-nephrotic proteinuria	22 (etiology not specified)	Eplerenone treatment significantly reduced proteinuria for up to 12 wk No difference in severe hyperkalemia incidence with eplerenone/ACEi versus ACEi
Finerenone				
*Filippatos et al. 2016 (ARTS-HF)^70^*	2b	Randomized, double-blind, controlled study of finerenone versus eplerenone in patients with worsening chronic heart failure and type 2 diabetes and/or CKD	1066 (35% had CKD without type 2 diabetes)	Both treatments induced a ≥30% decrease in NT-proBNP levels in similar proportions of patients Incidence of hyperkalemia at any time postbaseline was similar across groups
*Pitt et al. 2013 (ARTS)^69^*	2	Randomized, double-blind, placebo-controlled study of finerenone versus placebo and open-label spironolactone in patients with heart failure and reduced left ventricular ejection fraction and moderate CKD	392 (66% had nondiabetic CKD)	Finerenone was associated with significantly smaller mean increases in serum potassium concentration and lower incidences of hyperkalemia than spironolactone Finerenone also decreased the levels of BNP, NT-proBNP, and albuminuria at least as much as spironolactone Incidence of hyperkalemia with finerenone 5 or 10 mg significantly smaller versus spironolactone
KBP-5074				
*Bakris et al. 2021 (BLOCK-CKD)^67^*	2b	Randomized, double-blind, placebo-controlled study of developmental ns-MRA KBP-5074 in patients with stage 3b/4 CKD and uncontrolled hypertension	162 (included patients without diabetes)	KBP-5074 lowers BP with some risk of hyperkalemia Incidence of hyperkalemia slightly greater with 0.5 mg KBP-5074. No cases of severe hyperkalemia reported
**Diabetic (T2D) CKD**				
Finerenone				
*Bakris et al. 2020 (FIDELIO-DKD)^55^*	3	Randomized, double-blind, placebo-controlled study of finerenone versus placebo in patients with CKD associated with T2D (primary composite, kidney outcomes)	5674 (patients had T2D and CKD)	Lower risks of CKD progression and cardiovascular events with finerenone versus placebo Higher mean serum potassium with finerenone versus placebo and higher proportion of patients with serum potassium >5.5 mmol/L with finerenone versus placebo (21.7 versus 9.8%). Discontinuations of regimen because of hyperkalemia infrequent with finerenone (2.3%)
*Pitt et al. 2021 (FIGARO-DKD)^56^*	3	Randomized, double-blind, placebo-controlled study of finerenone versus placebo in patients with CKD associated with T2D (primary composite, cardiovascular outcomes)	7352 (patients had T2D and CKD)	Finerenone improved cardiovascular outcomes in patients with type 2 diabetes and stage 2–4 CKD with moderately elevated albuminuria or stage 1 or 2 CKD with severely elevated albuminuria versus placebo Incidence of hyperkalemia higher with finerenone versus placebo but none led to death, with few discontinuations of regimen (1.2% versus 0.4%) or hospitalization (0.6% versus 0.1%)

ACEi, angiotensin-converting enzyme inhibitor; ARB, angiotensin II receptor blocker; ARTS, mineralocorticoid receptor antagonist tolerability study; ARTS-HR, mineralocorticoid receptor antagonist tolerability study-heart failure; BNP, B-type natriuretic peptide; FIDELIO-DKD, finerenone in reducing kidney failure and disease progression in diabetic kidney disease; FIGARO-DKD; finerenone in reducing cardiovascular mortality and morbidity in diabetic kidney disease; LV, left ventricular; MRA, mineralocorticoid receptor antagonist; ns-MRA, nonsteroidal mineralocorticoid receptor antagonist; NT-proBNP, *N*-terminal pro–B-type natriuretic peptide; RAASi, renin-angiotensin-aldosterone system inhibitor; RCT, randomized controlled trial; T2D, type 2 diabetes; UACR, urine albumin-creatinine ratio.

a English publications only.

b The systematic review and meta-analysis by Bolignano *et al*.^62^ included one congress abstract (*Lv J, et al*. *NDT Plus 2009;2[Suppl 2]:ii1029 [abstract no: SA309]*) that could not be accessed independently for content verification and so is not included in this table.

## Published Data on MRAs in Nondiabetic Glomerular Disease

As described previously, nondiabetic glomerular disease is often overlooked as a feature of therapeutic research, and data on the long-term effects of MRAs on eGFR decline in patients with CKD without diabetes remain scarce. While studies have not typically focused on this patient population, some studies of the effect of MRAs on kidney outcomes have been conducted in mixed populations that included patients without diabetes (Table 2). For example, a Cochrane review published before the 2021 FDA approval of finerenone and consequently only including the steroidal MRAs (27 studies, plus one stated as ongoing) concluded that steroidal MRAs reduced proteinuria along with BP in patients with nondiabetic and diabetic CKD, although these drugs were associated with an increase in cases of hyperkalemia and gynecomastia.^62^ The findings from a recent systematic review and meta-analysis of ns-MRAs in patients with CKD, which included 11 trials and one pooled analysis, suggested that ns-MRAs provide a statistically beneficial effect on reducing the risk of the composite kidney outcome, as well as composite cardiovascular outcomes and all-cause mortality.^63^ It was also shown that ns-MRAs were associated with proteinuria remission and BP lowering.^63^ In addition, a pairwise meta-analysis of 26 studies concluded that MRA treatment could significantly reduce UACR in patients with CKD with or without diabetes. Importantly, the ns-MRAs (apararenone, esaxerenone, and finerenone) were associated with an overt UACR reduction without increasing serum potassium.^64^ An analysis of the kidney protective effects of spironolactone (plus RAASi) in patients with proteinuric nephropathies reported a drastic and sustained reduction in proteinuria and a trend toward slowing progression of kidney failure; approximately half of this study population did not have diabetes.^65^ In addition, a randomized placebo-controlled clinical trial of eplerenone in patients with stage 2 and 3 CKD (14.2% with diabetic nephropathy) revealed a significant preservative effect of MRA treatment on eGFR at 24 and 36 months.^66^ The benefits were most pronounced in patients with high plasma aldosterone levels. The results from a phase 2b study (BLOCK-CKD) of the developmental ns-MRA KBP-5074 showed a reduction in BP with some risk of hyperkalemia in individuals with advanced CKD; the study included patients without diabetes.^67^ In a retrospective study of low-dose spironolactone (12.5 mg/d) in 42 patients with GN (71.4% with IgA nephropathy) being treated with an ARB, proteinuria was significantly reduced over 3 months of treatment.^68^

Some data are available comparing steroidal and ns-MRAs in patients with CKD, including a proportion of patients who did not have diabetes. The results from a phase 2 RCT (minerAlocorticoid Receptor Antagonist Tolerability Study) that compared the efficacy and safety of finerenone versus spironolactone in patients with heart failure and reduced left ventricular ejection fraction and moderate CKD (two-thirds did not have diabetes) found that the nonsteroidal agent, finerenone, was at least as effective as spironolactone in decreasing biomarkers of hemodynamic stress but was associated with lower incidences of hyperkalemia and worsening kidney function.^69^ The results from a phase 2b RCT (mineralocorticoid receptor antagonist tolerability study-heart failure) that compared the efficacy and safety of finerenone versus eplerenone in patients with worsening chronic heart failure and type 2 diabetes and/or CKD (one-third with CKD without type 2 diabetes) found a similar 30% or greater decrease in *N*-terminal pro–B-type natriuretic peptide levels.^70^

There is interest in the potential benefits of combining MRAs with other renoprotective agents. A randomized, open-label crossover trial in patients with CKD revealed a robust additive UACR-lowering effect of combining the SGLT2i dapagliflozin with eplerenone (a clinically relevant albuminuria reduction of 53% after 4 weeks).^71^ Combining finerenone with an SGLT2i may also enhance finerenone's effects in CKD associated with type 2 diabetes, and the presence of an SGLT2i in the combination may potentially reduce hyperkalemic effects of finerenone.^28^ A phase 2 study (COmbinatioN effect of FInerenone anD EmpaglifloziN in participants with chronic kidney disease and type 2 diabetes using a UACR Endpoint study; NCT05254002) is ongoing to investigate this concept. Although a study focusing on cardiovascular outcomes with spironolactone in patients with early-stage (stage 3) CKD without diabetes (Spironolactone To Prevent Cardiovascular Events in Early Stage-CKD) was initiated, the trial was deemed unfeasible, mainly because of poor recruitment, and was terminated early.^72^ Figure 2 shows a treatment pathway for ns-MRA use/potential ns-MRA use in nondiabetic and diabetic CKD.

## Potential of ns-MRAs (Finerenone) in Nondiabetic CKD and Glomerular Disease

A common pathophysiologic feature of nondiabetic CKD and glomerular disease is immune overactivation or autoimmunity that contributes to kidney injury and dysfunction. As noted above, current treatments typically include immune suppression. For example, in IgA nephropathy, caused by deposition of immune complexes containing abnormal IgA antibody^73^ is associated with GN and progressive kidney disease. Here, immunosuppression is typically with glucocorticoids (Table 1^3^; KDIGO, 2021) (and other immunomodulatory therapy in development^74^), which aims to reduce the extent of glomerular inflammation. The ns-MRA finerenone has anti-inflammatory effects in the kidneys and heart, which are mediated through finerenone's selective inhibition of the MR (see section above on mode of action). Subanalyses from studies in patients with CKD associated with type 2 diabetes have shown that finerenone in combination with a RAASi can reduce the amount of the protein albumin (albuminuria) excreted in urine.^75,76^ Another anti-inflammatory effect of finerenone is switching of the proinflammatory M1 macrophage phenotype to the anti-inflammatory M2 phenotype because of finerenone-effected changes in gene expression through the MR in macrophages.^77^ Glucocorticoids also inhibit differentiation of macrophages into the M1 proinflammatory phenotype.^78,79^ Kidney fibrosis (including glomerulosclerosis) is a feature of advanced-stage CKD and ESKD (and of course sclerosis is a feature of FSGS)—preclinical models have shown that finerenone mediates a reduction in profibrotic gene expression (as well as proinflammatory gene expression)^31^ that leads to *in vivo* (mouse) kidney protective effects such as reduced myofibroblast numbers and collagen deposition.^80^ In phase 3 trials, these anti-inflammatory and antifibrotic effects of finerenone have translated into a decreased risk (versus placebo) of CKD progression and cardiovascular events in patients with CKD associated with type 2 diabetes (Table 2).^55,56^ In addition, the results from a retrospective review of outpatient data from a medical center in Japan suggest that finerenone may also be effective in patients who have nephrotic syndrome in addition to diabetic kidney disease because of an observed post-treatment reduction in albuminuria in these patients, although the results were not considered statistically significant.^81^ A prospective phase 3 clinical trial of finerenone in nondiabetic CKD (Finerenone In NonDiabetic-CKD) is ongoing (Table 3)—baseline results show that the most common causes of CKD were chronic GN (57%; of which IgA nephropathy was most common [26.3% total population]) and hypertensive/ischemic nephropathy (29.0%).^82^

**Table 3:** Ongoing clinical trials of nonsteroidal mineralocorticoid receptor antagonists for CKD/kidney impairment that may include patients without diabetes

Agent	Sponsor	Trial	Design	Status (April 2024)
AZD9977	AstraZeneca	MIRACLE; NCT04595370	Phase 2b trial in combination with dapagliflozin in patients with heart failure and CKD (including those with type 2 diabetes)	Complete (September 2023)
Balcinrenone	Nuremberg clinic/AstraZeneca	DapaBalci-Leap; NCT05884866	Phase 2, randomized, double-blind, double-dummy trial versus placebo or balcinrenone plus dapagliflozin in patients with CKD older than 50 yr (including those with type 2 diabetes)	Recruiting
Finerenone	Bayer	FIND-CKD; NCT05047263	Phase 3, randomized, double-blind, placebo-controlled trial+SOC in patients with nondiabetic CKD	Active, not recruiting
KBP-5074	KBP Biosciences	Clarion-CKD; NCT04968184	Phase 3, double-blind, placebo-controlled trial in patients with uncontrolled hypertension and moderate/severe CKD (diabetes not specified in inclusion/exclusion criteria)	Active, not recruiting

Source: ClinicalTrials.gov. FIND-CKD, finerenone in nondiabetic-CKD; SOC, standard of care.

## Considering the Safety of MRAS in Glomerular Disease

People with CKD have an increased risk of hyperkalemia, which can lead to serious issues such as cardiac arrhythmia episodes and sudden death.^83^ MRA treatments can potentially cause or worsen hyperkalemia in patients with kidney impairment^37,38^ (Table 2) and is a pressing safety concern of these drugs. As per prescribing information, eplerenone is contraindicated for treating hypertension in patients with a creatinine clearance <50 ml/min,^38^ and steroidal MRAs are not indicated for treatment of CKD with or without diabetes.^37,38^ A meta-analysis of data from studies of spironolactone and eplerenone has indicated that these agents conferred no significant risk of hyperkalemia in patients with nondiabetic proteinuric CKD, but this needs further investigation.^45^ The advantages of ns-MRAs include possibly lower risk of hyperkalemia (as seen with finerenone), having potency similar to spironolactone and greater than eplerenone, and greater selectivity for the MR, translating to its more specific anti-inflammatory and antifibrotic effects versus steroidal MRAs.^40^ It has been suggested that as finerenone increases potassium levels in a predictable way, patients at risk of hyperkalemia can be identified early in clinical practice and monitored for an easy management.^84^ Nonetheless, precautions are advised when prescribing finerenone.^36^ Serum potassium and eGFR should be measured in all patients before initiating treatment and should be monitored periodically during treatment, with the dose modified accordingly. Treatment should not be started if serum potassium is >5.0 mEq/L. Finally, more frequent monitoring may be necessary for patients at risk for hyperkalemia, including those on concomitant medications that impair potassium excretion or increase serum potassium.^36^

As noted, potassium binders such as patiromer and sodium zirconium cyclosilicate are available to manage hyperkalemia.^28,85^ The ongoing MorphCKD trial is exploring the feasibility of adding patiromer to allow for improved renin-angiotensin-aldosterone system blockade (including use of an MRA), thereby reducing albuminuria in patients with albuminuric CKD where full treatment might otherwise be limited by hyperkalemia.^86^ As noted in the KDIGO 2022 guidelines, “hyperkalemia associated with the use of an ACEi or ARB can often be managed by measures to reduce serum potassium levels rather than decreasing the dose or stopping the ACEi or ARB immediately”; these measures include a low potassium diet and the use of diuretics and potassium binders.^28^

## Ongoing Clinical Trials of Novel MRAs in CKD

Several novel ns-MRAs are in clinical development for CKD/kidney impairment; however, clinical trials generally also include patients with diabetes. These are listed in Table 3.

## Conclusions

MRAs have antiproteinuric effects. The approval of the ns-MRA finerenone for use in CKD associated with type 2 diabetes represents a new phase in management of cardiorenal conditions, marking an improved risk−benefit profile of MRA agents and thus a wider therapeutic potential. Nondiabetic glomerular disease has typically been underrecognized and understudied relative to diabetic nephropathies. However, ns-MRAs may offer benefits to patients without diabetes and thus present a wide-ranging opportunity to reduce the progression of CKD from a spectrum of glomerular diseases.

## Supplementary Material
