# Supplementary material for: Potential Role of Mineralocorticoid Receptor Antagonists in Nondiabetic Chronic Kidney Disease and Glomerular Disease
Source: Clin J Am Soc Nephrol. 2024 Jul 22;19(11):1499–512. doi: 10.2215/CJN.0000000000000540 (PMC11556932; doi:10.2215/CJN.0000000000000540)
Supplement: Supplementary file 1 [file cjasn-19-1499-s001.pdf]

ASN American Society of Nephrology JASN Journal of the American Society of Nephrology KIDNEY360 Accessing Our World From Every Angle CJASN Clinical Journal of the American Society of Nephrology

ASN Journal Disclosure Form

As per ASN journal policy, I have disclosed any financial relationships or commitments I have held in the past 36 months as included below. I have listed my Current Employer below to indicate there is a relationship requiring disclosure. If no relationship exists, my Current Employer is not listed.

J. Radhakrishnan reports the following:
Employer: Columbia University Medical Center; Consultancy: Reistone Biopharma; Sanofi Genzyme; Equillium Bio; Aurinia Pharmaceuticals; Reata Pharmaceuticals; Travere Therapeutics; Angion Biomedica, Ani Pharmaceuticals, Novartis, Goldfinch Bio, Chinook, Calliditas, Alexion,; Research Funding: Mineralys Therapeutics, Vertex pharmaceuticals; Honoraria: Reistone Biopharma; Sanofi Genzyme; Equillium Bio; Aurinia Pharmaceuticals; Reata Pharmaceuticals; Travere Therapeutics; Angion Biomedica, Ani Pharmaceuticals, Novartis, Goldfinch Bio, Chinook, Calliditas, Glaxo Smith Kline; Advisory or Leadership Role: Associate Editor: Kidney International; Editor: Kidney International Reports, KDIGO Glomerular Disease Guideline Committee; and Speakers Bureau: Amgen.

I understand that the information above will be published within the journal article, if accepted, and that failure to comply and/or to accurately and completely report the potential financial conflicts of interest could lead to the following: 1) Prior to publication, article rejection, or 2) Post-publication, sanctions ranging from, but not limited to, issuing a correction, reporting the inaccurate information to the authors' institution, banning authors from submitting work to ASN journals for varying lengths of time, and/or retraction of the published work.

Name: Jai Radhakrishnan
Manuscript ID: CJASN-2024-000303R2
Manuscript Title: Potential Role of Mineralocorticoid Receptor Antagonists in Nondiabetic Chronic Kidney Disease and Glomerular Disease
Date of Completion: June 25, 2024
Disclosure Updated Date: June 25, 2024

ASN American Society of Nephrology | JASN Journal of the American Society of Nephrology | KIDNEY360 Accessing Our World From Every Angle | CJASN Clinical Journal of the American Society of Nephrology

ASN Journal Disclosure Form

As per ASN journal policy, I have disclosed any financial relationships or commitments I have held in the past 36 months as included below. I have listed my Current Employer below to indicate there is a relationship requiring disclosure. If no relationship exists, my Current Employer is not listed.

T. Zachariah reports the following:
Employer: Columbia University Medical Center

I understand that the information above will be published within the journal article, if accepted, and that failure to comply and/or to accurately and completely report the potential financial conflicts of interest could lead to the following: 1) Prior to publication, article rejection, or 2) Post-publication, sanctions ranging from, but not limited to, issuing a correction, reporting the inaccurate information to the authors' institution, banning authors from submitting work to ASN journals for varying lengths of time, and/or retraction of the published work.

Name: Teena Zachariah
Manuscript ID: CJASN-2024-000303R2
Manuscript Title: Potential Role of Mineralocorticoid Receptor Antagonists in Nondiabetic Chronic Kidney Disease and Glomerular Disease
Date of Completion: July 8, 2024
Disclosure Updated Date: July 8, 2024
